# Influence of Substituent Chain Branching on the Transfection Efficacy of Cyclopropenium-Based Polymers

**DOI:** 10.3390/polym9030079

**Published:** 2017-02-24

**Authors:** Spencer D. Brucks, Jessica L. Freyer, Tristan H. Lambert, Luis M. Campos

**Affiliations:** Department of Chemistry, Columbia University, 3000 Broadway, New York, NY 10027, USA; sdb2147@columbia.edu (S.D.B.); jlf2176@columbia.edu (J.L.F.); tl2240@columbia.edu (T.H.L.)

**Keywords:** gene delivery, polyelectrolyte, nonviral vectors, structure-property relationships

## Abstract

The realization of gene therapy relies on the development of delivery vectors with high efficiency and biocompatibility. With a multitude of structures accessible, the core challenge is precisely tuning vector structure to probe and optimize structure–property relationships. Employing a modular strategy, two pairs of cationic polymers based on the trisaminocyclopropenium (TAC) ion were synthesized where the substituents differ in the degree of alkyl chain branching. All TAC-based polymers exhibited higher transfection efficiencies than the untreated controls, with variable in vitro toxicities. Considering both cytotoxicity and transfection efficacy, an optimal nonviral vector was identified. Our studies highlight the importance of exercising precise control over polymer structure, both in terms of backbone identity and substituent nature, and the necessity of a robust, modular platform from which to study them.

## 1. Introduction

The fundamental challenge of gene therapy is the design of efficient, biocompatible delivery vectors [1,2,3]. Since the concept of gene delivery was first introduced, both viral and nonviral options have been explored, with each presenting their own benefits and drawbacks [1,4]. While viral technologies demonstrate high gene expression, their clinical translation has been limited by immunogenicity, lack of selectivity, and synthetic difficulty [2,5]. On the contrary, nonviral vectors elicit a reduced immunogenic response, but thus far have shown lower delivery efficiencies. The development of an optimized nonviral vector, balancing safety with efficiency, thus remains a critical goal to the realization of gene therapy [1,2,6].

Cationic polymers are one of the most commonly studied nonviral vectors due to their high stability and capacity to tune macromolecular composition and architecture [7,8]. Linear polyethylenimine (PEI) was the first cationic polymer observed to rapidly complex negatively charged nucleic acids into polyplexes [9]. In the subsequent decades, there has been a great proliferation of cationic structures shown to bind and transfect nucleic acids. In addition to increasing the library of cationic polymers available, these research efforts have also uncovered some design principles towards optimizing polymeric structure for transfection [1,7]. In general, lower molecular weight polymers demonstrate lowered cytotoxicity than their high molecular weight counterparts [10,11,12,13], and increasing the degree of polymer chain branching appears to heighten both transfection efficacy and toxicity [14,15,16,17]. Wang and coworkers have recently highlighted the great potential of branched and dendritic polymer topologies to enhance transfection efficiency [18,19,20,21]. With the advent of controlled polymerization techniques, the challenge has now become fine-tuning polymer structure, size, and architecture to probe detailed structure–property relationships among the various polymers accessible.

We recently reported a modular platform to functionalize polymers of various sizes and architectures with a host of bis(dialkylamino)cyclopropenium chloride (BACCl) derivatives [22]. In an efficient post-polymerization click reaction, polymers bearing pendent or main-chain secondary amines were quantitatively transformed into the aromatic trisaminocyclopropenium (TAC) ion. We found that some of the resulting TAC polymers were biocompatible and efficient transfection agents and, furthermore, the ability to precisely tune polymer structure had significant effects on macromolecular properties, including transfection efficiency.

**Figure 1:**
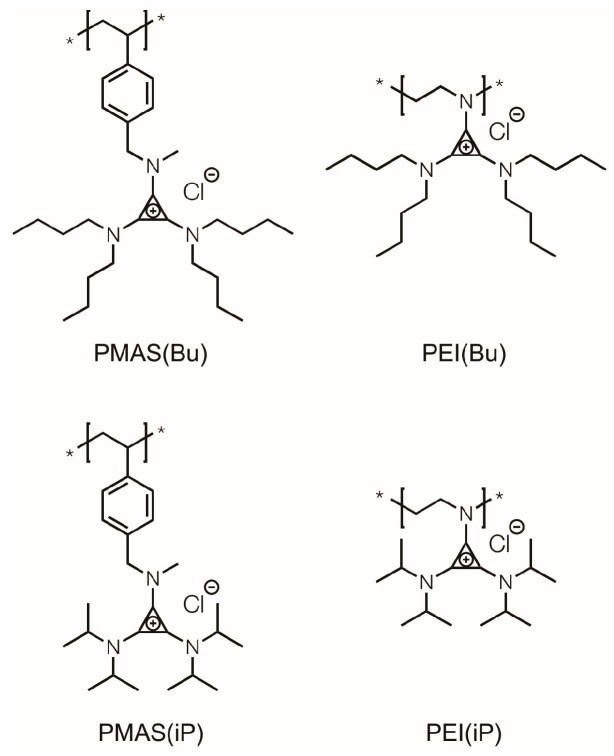
Trisaminocyclopropenium (TAC) polymer structures examined for biocompatibility and transfection efficacy. PEI: polyethylenimine; PMAS: poly(methylaminostyrene); Bu: *n*-butyl; iP: isopropyl.

Herein, we extend these results by probing the effect of the substituted alkylamino chain’s degree of branching on cytotoxicity and transfection efficacy. Pendent moieties have been shown to modify the structure and stability of polymer–DNA polyplexes to facilitate cellular release, with several reports exploring the effects of fine tuning a substituent alkyl chain length [23,24,25]. However, comparably few studies have investigated the nature of branching within an alkyl group and its effect on transfection efficacy. As our platform is amenable to a wide variety of BACCl derivatives, we synthesized two that would directly compare the branching of the alkyl chain: *n*-butyl (Bu; BACBu) and isopropyl (iP; BACiP). Employing these ionic liquids in the post-polymerization functionalization of two polymeric backbones, poly(methylaminostyrene) (PMAS) and polyethylenimine (PEI), furnished a total of four polymers that we investigated as nonviral vectors (Figure 1). We found that there exist important synergies between the polymeric backbone and the nature of the substituent, and that the ability to simultaneously manipulate both is instrumental for the optimization of efficient nonviral gene delivery vectors.

## 2. Materials and Methods

### 2.1. Materials

All materials were purchased from Sigma-Aldrich (St. Louis, MO, USA) and were used without further purification except as noted below. Deuterated solvents used for NMR spectroscopy were purchased from Thermo Fisher Scientific (Waltham, MA, USA). Spectrum Labs dialysis bags were purchased from VWR (Radnor, PA, USA). Organic solutions were concentrated by use of a Buchi (New Castle, DE, USA) rotary evaporator.

### 2.2. Procedure for the Synthesis of 1,2-Bis(dibutylamino)-3-chlorocyclopropenium Chloride (BACBu)

#### 2.2.1. Synthesis of 2,3-Bis(dibutylamino)-1-cyclopropenone

This procedure was performed at ambient conditions. Dibutylamine (24.5 g, 190 mmol, 8.0 equiv) was slowly added to a solution of pentachlorocyclopropane (5.0 g, 23.6 mmol, 1.0 equiv) in CH_2_Cl_2_ (250 mL) in a 1 L round-bottom flask at 0 °C. The solution turned orange, and was allowed to warm to room temperature with stirring overnight. The reaction mixture was washed with 1 M HCl (3 × 100 mL), deionized (DI) water (1 × 100 mL), and brine (1 × 100 mL) dried over magnesium sulfate, and concentrated in vacuo to yield a crude orange solid. The solid was dissolved in *tert*-butanol (50 mL) and to this was added potassium hydroxide (10 g, 178 mmol) in DI water (15 mL). The solution was heated at 70 °C for 2 h, and then water was removed by rotary evaporation. The resulting solid was dissolved in CH_2_Cl_2_ and filtered to remove salt. The organic solution was dried with anhydrous magnesium sulfate, and concentrated in vacuo to a crude yellow oil. The crude material was purified by silica gel chromatography (100% EtOAc; 5% MeOH in CH_2_Cl_2_) to yield the title product as an orange solid (2.19 g, 7.08 mmol, 30% two-step yield). ^1^H NMR (400 MHz, CDCl_3_) δ 3.16 (t, 8H, NCH_2_CH_2_CH_2_CH_3_), 1.59 (m, 8H, NCH_2_CH_2_CH_2_CH_3_), 1.34 (m, 8H, NCH_2_CH_2_CH_2_CH_3_), 0.94 (t, 12H, NCH_2_CH_2_CH_2_CH_3_).

#### 2.2.2. Synthesis of 1,2-Bis(dibutylamino)-3-chlorocyclopropenium Chloride (BACBu)

Oxalyl chloride (0.09 mL, 0.9 mmol, 2.0 equiv) was slowly added to a solution of 2,3-bis(dibutylamino)-1-cyclopropenone (0.150 g, 0.45 mmol, 1.0 equiv) in dry CH_2_Cl_2_ (5 mL) at 0 °C under argon. The solution was warmed to room temperature and allowed to react for 1 h. The solution was then concentrated in vacuo to yield the title product as a brown liquid in quantitative yield. ^1^H NMR (400 MHz, CDCl_3_) δ 3.64 (t, 4H, NCH_2_CH_2_CH_2_CH_3_), 3.50 (t, 4H, NCH_2_CH_2_CH_2_CH_3_), 1.76 (m, 4H, NCH_2_CH_2_CH_2_CH_3_), 1.66 (m, 4H, NCH_2_CH_2_CH_2_CH_3_), 1.40 (m, 8H, NCH_2_CH_2_CH_2_CH_3_), 0.99 (t, 12H, NCH_2_CH_2_CH_2_CH_3_).

### 2.3. Synthesis of PMAS(Bu)

The procedure was performed open to the atmosphere. Poly(methylaminostyrene) (PMAS) (50 mg, 0.3 mmol, 1 equiv, DP: 50, *M*_n_: 7400, *M*_w_: 8300, Đ: 1.08), synthesized according to previously reported procedures [22], was dissolved in CHCl_3_ (8 mL) in a scintillation vial equipped with a stir bar. To the vial was added *N*,*N*-diisopropylethylamine (115 mg, 0.9 mmol, 3 equiv) and BACBu (160 mg, 0.46 mmol, 1.5 equiv). The reaction mixture was allowed to stir at 65 °C for 3 h. The resulting solution was concentrated in vacuo, dissolved in minimum acetone and precipitated once into ethyl acetate at −78 °C. The resulting powder was dissolved in methanol and transferred to a 3.5k MWCO Spectrum Labs dialysis bag and dialyzed against methanol followed by concentration under vacuum to yield a pale brown powder (60 mg, 42% yield). ^1^H NMR (500 MHz, CDCl_3_) δ 7.18–6.07 (b, 200H, ArH), 4.99–4.39 (b, 100H, ArCH_2_N), 3.78–2.91 (b, 575H, NCH_3_, NCH_2_CH_2_CH_2_CH_3_), 1.98–1.58 (b, 400H, NCH_2_CH_2_CH_2_CH_3_) 1.39–1.08 (b, 650H, NCH_2_CH_2_CH_2_CH_3_, ArCHCH_2_) 1.02–0.63 (b, 600H, NCH_2_CH_2_CH_2_CH_3_).

### 2.4. Synthesis of PEI(Bu)

This procedure was performed open to the atmosphere. Linear 25k polyethyleneimine (20 mg, 0.46 mmol, 1 equiv) was dissolved in CHCl_3_ (8 mL) in a scintillation vial equipped with a stir bar. To the vial was added *N,N*-diisopropylethylamine (180 mg, 1.4 mmol, 3 equiv) and BACBu (250 mg, 0.70 mmol, 1.5 equiv). The reaction mixture was allowed to stir at 65 °C for 3 h. The resulting solution was concentrated in vacuo and precipitated once into ethyl acetate at −78 °C. The resulting powder was dissolved in methanol and transferred to a 3.5k MWCO Spectrum Labs dialysis bag and dialyzed against methanol. The resulting solution was concentrated vacuum to yield a yellow-brown powder (25 mg, 15% yield). ^1^H NMR (400 MHz, CDCl_3_) δ 4.45–3.15 (b, 7000H, CH_2_CH_2_N)_581_, NC**H_2_**CH_2_CH_2_CH_3_) 1.71–1.55 (b, 3500H, NCH_2_CH_2_CH_2_CH_3_), 1.43–1.22 (b, 5400H, NCH_2_CH_2_CH_2_CH_3_), 1.00–0.90 (b, 6200H, NCH_2_CH_2_CH_2_CH_3_).

### 2.5. Synthesis of 1,2-Bis(diisopropylamino)-3-chlorocyclopropenium Chloride (BACiP), PMAS(iP), and PEI(iP)

The preparations of BAC(iP) and the subsequently derivatized polymers have been previously reported [22,26].

### 2.6. Cell Culture

HEK-293T cells (American Type Culture Collection, Manassas, VA, USA) were grown in Dulbecco’s Modified Eagle Medium (DMEM) with l-glutamine (Gibco, Grand Island, NY, USA) supplemented with 10% fetal bovine serum (FBS) (Atlanta Biologicals, Flowery Branch, GA, USA) and 1% penicillin/streptomycin (Gibco, Grand Island, NY, USA). Cultures were incubated in humidified tissue incubators at 37 °C and 5% CO_2_.

### 2.7. Cell Viability

Trypan blue dye exclusion cell counting was performed in triplicate with an automated cell counter (ViCell, Beckman-Coulter, Brea, CA, USA). Cell viability under experimental conditions is reported as a percentage relative to untreated cells.

### 2.8. Cell Transfection and Luciferase Expression

HEK-293T cells were seeded on 12-well plates at a density of 5 × 10^4^ cells/well 24 h prior to transfection. The media was then evacuated, replaced with fresh, and supplemented with polymer–pDNA polyplexes. Polyplexes were prepared by adding polymer solutions in RNase-free water to 3 µg of plasmid DNA (pDNA) (gWiz-Luciferase, Aldevron, Fargo, ND, USA) at indicated loadings, and vortexing at 1500 rpm for 3 min at room temperature. After 48 h of incubation, cell viability was measured, and cells were re-plated on 96-well plates at a density of 5 × 10^3^ cells/well. After 24 h of incubation, cells were analyzed for luciferase activity according to the manufacturer’s protocol. Briefly, cells were rinsed with phosphate-buffered saline (PBS) and lysed with 20 µL/well 1 × Cell Lysis Buffer (Promega, Madison, WI, USA). To the cell lysates was added 100 µL/well of Luciferase Assay Reagent (Promega) and the light produced was measured immediately on a plate reader (PerkinElmer, Waltham, MA). Results were expressed as relative light units (RLU) normalized to cell counts, with error bars showing the standard deviation of triplicate measurement.

### 2.9. Hydrodynamic Size and Zeta Potential Measurement

Polyplex size and zeta potential were measured on a Malvern Zetasizer Nano ZS (Malvern Instruments, Malvern, UK). For all measurements, polyplexes were diluted 1:100 in Milli-Q water at neutral pH. The reported diameters are the average of three measurements, where each measurement comprises at least 10 acquisitions, and the zeta potential was calculated according to the Smoluchowski approximation.

### 2.10. Gel Electrophoresis Shift Assay

Polyplexes were prepared at different weight ratios by adding 10 µL of polymer in Milli-Q H_2_O to 10 µL of pDNA (5 ng/µL), and vortexing at 1500 rpm for 3 min at room temperature. To the polyplex solution was then added 2 µL of loading dye, for a total volume of 22 µL, which was subsequently added to the well. Agarose gels were prepared as 1 wt % in tris-acetate EDTA (TAE) buffer with 2 µL ethidium bromide and run at 100 V for 20 min. Gels were visualized under UV illumination at 365 nm.

## 3. Results and Discussion

### 3.1. Cationic Polymer Synthesis

The candidate nonviral vectors were synthesized from neutral parent precursor polymers in a post-polymerization functionalization strategy. For this study, we employed two parent polymers—PMAS (7.5 kg·mol*^−^*^1^), synthesized according to literature [22], and commercially available PEI (25 kg·mol^–1^; linear)—to compare the effects of backbone structure. Both were subsequently transformed into TAC derivatives by reaction with a BACCl salt, in a “click” conjugation reaction proceeding under mild conditions with stoichiometric amounts of reactants. The cationic nature of cyclopropenium-based polymers prevents their characterization by gel permeation chromatography [26], so PMAS was shown to have a narrow dispersity less than 1.1 (Supplementary Materials, Figure S1), and complete functionalization was confirmed by NMR.

We elected to study two BACCl structures comprising dialkylamino substituents differing in the degree of branching, and thereby “floppiness”, as well as hydrophobicity, as the Bu-derivatized polymers have one more carbon. Complete functionalization of both BAC(Bu), containing n-butyl substituents, and BAC(iP), containing isopropyl substituents, was confirmed by proton nuclear magnetic resonance spectroscopy. Quantitatively functionalizing polymers holds effects of dispersity and degree of polymerization constant, permitting direct comparisons of subtle structural changes on macromolecular properties.

### 3.2. Biocompatibility Studies

Careful engineering of cationic polymers is necessary to enable permeation of the cell membrane without reducing overall viability. Studies have shown that cationic polymers form pores in the cell membrane to mediate entry, but they also reduce overall cell viability [27]. Pore formation typically involves intercalation of the cell membrane’s lipid bilayer by aliphatic groups on the delivery agent; thus, tuning TAC’s alkyl substituents represents an opportunity to promote cell entry while minimizing cell death associated with membrane destabilization. Therefore, to probe the impact of alkyl chain conformation on cell viability and transfection efficiency, the series of TAC-functionalized polymers were assessed and compared as vectors via cytotoxicity assays and luciferase transfection experiments in HEK-293T cells.

**Figure 2:**
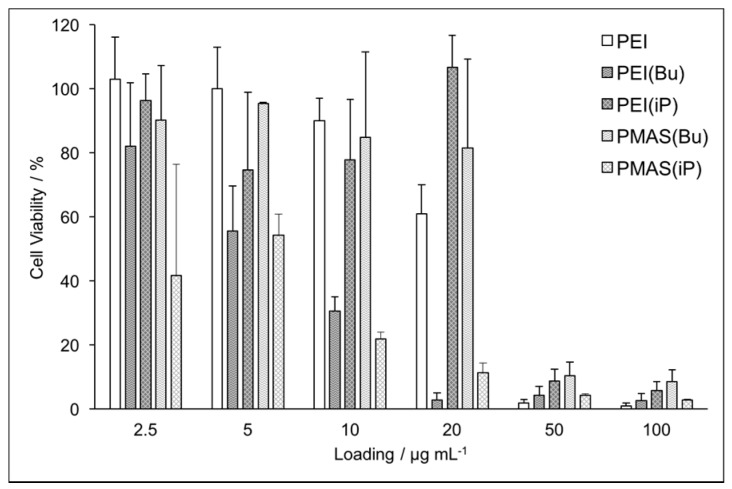
Biocompatibility of TAC-based polymers at various doses in HEK-293T cells following 48 h incubation. Viability is measured by trypan blue dye exclusion and normalized to untreated cells. Error bars show the standard deviation of triplicate measurement.

All four homopolymers were highly water-soluble, permitting their condensation with an aqueous solution of plasmid DNA (pDNA) containing the firefly luciferase reporter gene. Combining the polymers at varied loadings with a fixed amount of pDNA, and subsequently incubating in cells for 2 d, revealed the polymers’ biocompatibility as a function of loading. We found that PEI(iP) and PMAS(Bu) were the most biocompatible with high cell viabilities through loadings of 20 µg·mL*^−^*^1^ (Figure 2). Surprisingly, their counterparts, PEI(Bu) and PMAS(iP), exhibited notable toxicity at all loadings tested. All TAC-derived polymers here were highly toxic at loadings of 50 µg·mL*^−^*^1^ and greater, similar to linear PEI. This stands in contrast to our previous work, where polymers with more rigid amino substituents on the TAC ion were still viable in this regime [22]. Thus, rigidity or flexibility of substituent chains stand as an important parameter to understand for optimal gene transfection. These results suggest there is a complex interaction between a polymer backbone and its substituent in the design of biocompatible gene delivery vectors.

### 3.3. DNA Binding and In Vitro Gene Transfection

In order to affect transfection, candidate gene delivery vectors must condense nucleic acids into a polyplex, permeate the cell membrane, escape the endosome, and release their payload [27]. We found that fine tuning the substituent chain branching had a dramatic influence on delivery efficacy. In order to assess the amount of polymer necessary to completely condense pDNA into a polyplex, gel electrophoresis shift assays were performed (Figures S2–S5). While all polymers were able to fully bind the pDNA by a weight ratio of 3:33:1, PEI(iP) was the most efficient, binding at a weight ratio of only 0.83:1. This corresponds to the lowest polymer loading tested for either biocompatibility or transfection, and less than 1 TAC unit per phosphate anion of pDNA (Tables S1 and S2). However, binding efficiency is not a clear indicator of delivery efficiency, as too favorable an interaction can be detrimental for eventual release of the genetic material.

**Figure 3:**
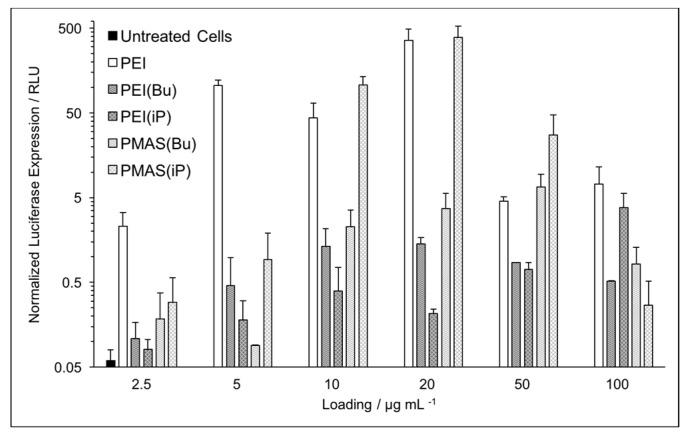
Luciferase expression in HEK-293T cells transfected with pDNA containing the firefly luciferase reporter gene using TAC polymers. Luciferase expression is measured after 48 h incubation with specified polymer loadings (all with pDNA loadings of 3 µg·mL^−1^) and normalized by cell count. Error bars show the standard deviation of triplicate measurement.

Luciferase expression assays revealed a significant dependence on the nature of the amino substituent and polymer backbone for successful gene delivery. While all TAC-based polymers transfected pDNA significantly better than the untreated controls, PMAS(iP) demonstrated the highest transfection efficacy (Figure 3). As is the case with unmodified linear PEI, successful delivery of intact pDNA to cells comes at the cost of significant cytotoxicity. By contrast, the nontoxic PEI(iP) demonstrated a much lower luciferase activity. Interestingly, PEI(iP) was the most efficient at compacting pDNA into a polyplex, suggesting that it binds nucleic acids too strongly and never releases its payload. Converting either of the polymer backbones into a TAC bearing n-butyl chains seemed to yield successful nonviral vectors capable of both binding and slowly releasing pDNA. This could potentially be attributed to critical destabilization of the cell and endosomal membranes due to the long, flexible alkyl substituents. As none of the TAC polymers examined here contain any protonatable centers, it is unlikely that polyplexes escape the endosome via a proton sponge mechanism [24]. At their optimal loadings, both PEI(Bu) and PMAS(Bu) demonstrated two orders-of-magnitude improvement over untreated control cells. Taken together with the cytotoxicity and pDNA-binding data, we conclude that amongst this family of TAC polymers, PMAS(Bu) is the most potent nonviral vector.

### 3.4. Hydrodynamic Size and Zeta Potential Measurements

Dynamic light scattering determined the hydrodynamic diameter (D_H_) and zeta potential (ζ) of the polyplexes at their optimal loading for transfection efficacy (Table 1). All four cationic polymers formed stable polyplexes of small sizes and highly positive charge. The polyplexes all exhibit a hydrodynamic diameter in the size regime considered optimal for successful gene transfection. Notably, the polymers modified with BAC(iP) resulted in more positively charged polyplexes than those with BAC(Bu), which could be a result of enhanced hydrophobic screening of the charge by the longer, flexible n-butyl chains [20,28]. These data suggest the dramatic differences in cytotoxicity and transfection efficacy between the four TAC-based polymers are likely a result of structural variations rather than significant size or surface charge differences.

## 4. Conclusions

We have shown that fine-tuning polymer structure can have dramatic effects on macromolecular properties—in particular, cytotoxicity and gene delivery. Beginning with two discrete parent polymers, we synthesized two pairs of TAC-based polymers differing solely in alkyl substituent identity, branching, and polymer backbone. While none of the examined polymers surpass linear PEI in transfection efficiency, our results demonstrate that there is an important interplay between polymer backbone and substituent structure, and that both must be carefully considered in the design of nonviral vectors. Amongst our examined TAC-based polymers, PMAS(Bu) exhibited the best balance of biocompatibility, efficient DNA binding, and transfection efficacy. While hydrophobic modifications of nonviral vectors are frequently reported to promote transfection, our results demonstrate that a careful balance of hydrophobicity and substituent flexibility must be achieved for optimal gene delivery. Importantly, this work exemplifies that design of transfection reagents demands precise control over all aspects of polymer structure and a robust, modular platform from which to study them. Only through these kinds of systematic studies can an optimized nonviral vector to achieve successful gene delivery be developed.

## Figures and Tables

**Table 1:** Characterization of transfection agents and polyplexes at optimal transfection efficacy.

Transfection agent ^1^	MM ^2^ (kDa)	Charge Ratio ^3^	D_H_ (nm)	*ζ* Potential (mV)
PEI(Bu)	215	6:1	110 ± 40	45 ± 7
PEI(iP)	182	35:1	100 ± 40	58 ± 5
PMAS(Bu)	25	12:1	150 ± 50	31 ± 8
PMAS(iP)	21	5:1	160 ± 40	44 ± 6

^1^ Polyplexes of polymers at the loading corresponding to highest transfection efficacy in Figure 3. ^2^ Molecular mass of transfection agent, calculated based on commercial linear 25k PEI; for PMAS(R) materials, PMAS was measured by gel permeation chromatography (GPC) calibrated using polystyrene (PS) standards of narrow dispersity, then calculated for the corresponding TAC group. ^3^ Ratio of TAC to phosphate anions.

## Supplementary Materials

The following are available online at www.mdpi.com/2073-4360/9/3/79/s1, Figure S1: GPC trace of poly(methylaminostyrene), the parent polymer for both PMAS(Bu) and PMAS(iP); Figure S2: Gel electrophoresis shift assay of pDNA polyplexes formed with PMAS(Bu) at the indicated polymer: pDNA weight ratios. All pDNA is bound in polyplexes at a weight ratio of 1.66 PMAS(Bu): 1 pDNA; Figure S3: Gel electrophoresis shift assay of pDNA polyplexes formed with PEI(Bu) at the indicated polymer:pDNA weight ratios. All pDNA is bound in polyplexes at a weight ratio of 3.33 PEI(Bu): 1 pDNA; Figure S4: Gel electrophoresis shift assay of pDNA polyplexes formed with PMAS(iP) at the indicated polymer:pDNA weight ratios. All pDNA is bound in polyplexes at a weight ratio of 1.66 PMAS(iP): 1 pDNA; Figure S5: Gel electrophoresis shift assay of pDNA polyplexes formed with PEI(iP) at the indicated polymer:pDNA weight ratios. All pDNA is bound in polyplexes at a weight ratio of 0.83 PEI(iP): 1 pDNA; Table S1: Polymer loading and pDNA weight ratios for gel electrophoresis and transfection experiments; Table S2: Conversion of weight ratios to charge ratios for each tested polymer.
