# Supplementary material for: Influence of Substituent Chain Branching on the Transfection Efficacy of Cyclopropenium-Based Polymers
Source: Polymers (Basel). 2017 Feb 24;9(3):79. doi: 10.3390/polym9030079 (PMC6431972; doi:10.3390/polym9030079)
Supplement: Supplementary file 1 [file polymers-09-00079-s001.pdf]

Supplementary Materials: Influence of Substituent Chain Branching on the Transfection Efficacy of Cyclopropenium-Based Polymers

Spencer D. Brucks, Jessica L. Freyer, Tristan Lambert and Luis M. Campos

Table S1. Polymer loading and plasmid DNA (pDNA) weight ratios for gel electrophoresis and transfection experiments.

Weight ratios [1]	0.83:1	1.66:1	3.33:1	6.66:1	16.7:1	33.3:1
Gel (ng) [2]	42	83	167	333	835	1665
Transfection (μg) [3]	2.5	5	10	20	50	100

[1] Weight ratio is given as polymer:pDNA. [2] Mass of polymer added for gel electrophoresis shift assay for indicated weight ratio. [3] Mass of polymer added for biocompatibility and transfection experiments for indicated weight ratio.

Table S2. Conversion of weight ratios to charge ratios for each tested polymer.

Weight ratios[1]	0.83:1	1.66:1	3.33:1	6.66:1	16.7:1	33.3:1
PEI(Bu)	0.74:1	1.48:1	2.95:1	5.9:1	14.8:1	29.5:1
PEI(iP)	0.87:1	1.74:1	3.48:1	6.97:1	17.5:1	34.8:1
PMAS(Bu)	0.58:1	1.15:1	2.31:1	4.6:1	11.6:1	23.1:1
PMAS(iP)	0.66:1	1:31:1	2.62:1	5.25:1	13.1:1	26.2:1

[1] Weight and charge ratios are given as polymer:pDNA.

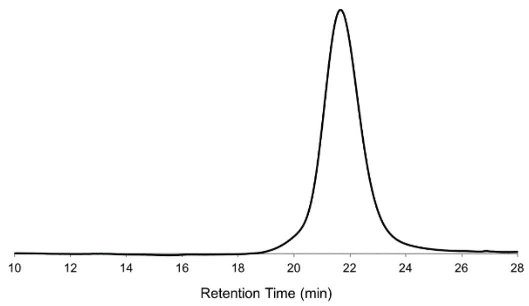

Figure S1. Gel permeation chromatography (GPC) trace of poly(methylaminostyrene), the parent polymer for both PMAS(Bu) and PMAS(iP).

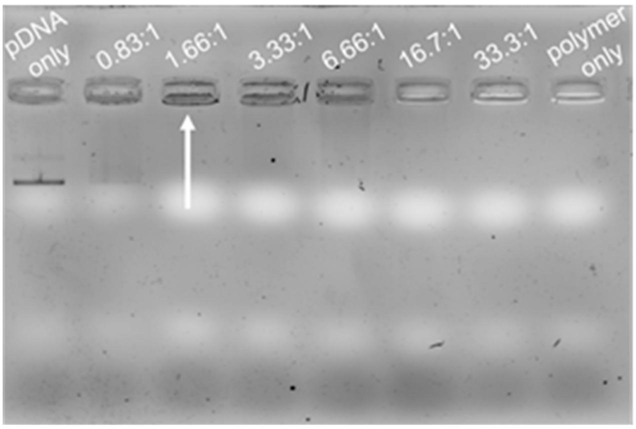

Figure S2. Gel electrophoresis shift assay of pDNA polyplexes formed with PMAS(Bu) at the indicated polymer:pDNA weight ratios. All pDNA is bound in polyplexes at a weight ratio of 1.66 PMAS(Bu):1 pDNA.

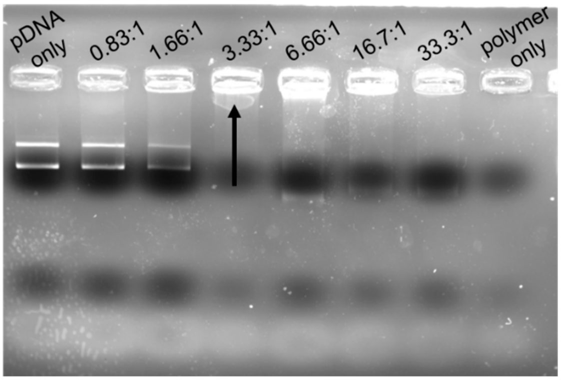

Figure S3. Gel electrophoresis shift assay of pDNA polyplexes formed with PEI(Bu) at the indicated polymer:pDNA weight ratios. All pDNA is bound in polyplexes at a weight ratio of 3.33 PEI(Bu):1 pDNA.

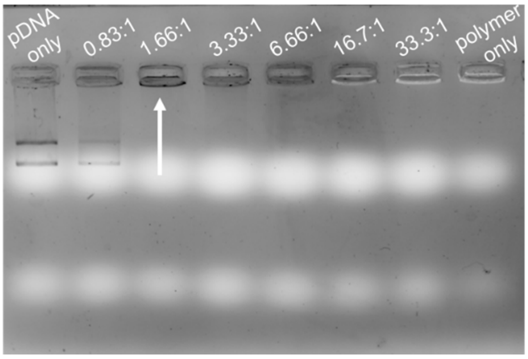

Figure S4. Gel electrophoresis shift assay of pDNA polyplexes formed with PMAS(iP) at the indicated polymer:pDNA weight ratios. All pDNA is bound in polyplexes at a weight ratio of 1.66 PMAS(iP):1 pDNA.

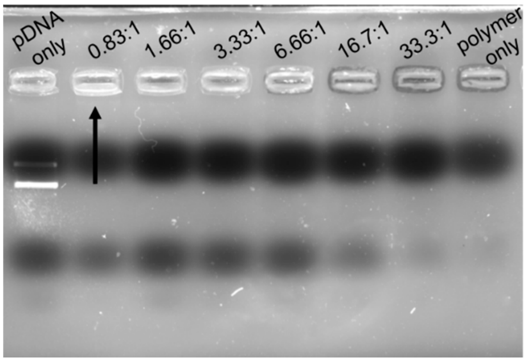

Figure S5. Gel electrophoresis shift assay of pDNA polyplexes formed with PEI(iP) at the indicated polymer:pDNA weight ratios. All pDNA is bound in polyplexes at a weight ratio of 0.83 PEI(iP):1 pDNA.
